# Herpes Zoster Radiculomyelitis With Aquaporin-4 Antibodies: A Case Report and Literature Review

**DOI:** 10.3389/fneur.2020.585303

**Published:** 2020-11-23

**Authors:** Hiroto Eguchi, Haruka Takeshige, Sho Nakajima, Masayoshi Kanou, Asuka Nakajima, Atsuto Fuse, Jiro Fukae, Hideto Miwa, Yasushi Shimo

**Affiliations:** ^1^Department of Neurology, Nerima Hospital of Juntendo University School of Medicine, Tokyo, Japan; ^2^Department of Neurology, Juntendo University School of Medicine, Tokyo, Japan

**Keywords:** herpes zoster, aquqporin-4 immunoglobulin G, myelitis, neuromyelitis optica spectrum disorder, case report

## Abstract

**Background:** The relationship between varicella-zoster virus (VZV)-associated myelitis and aquaporin-4 immunoglobulin-G (AQP4-IgG) remains unknown.

**Case Report:** We report a case of acute radiculomyelitis with longitudinal extensive hyperintensity signals traversing the brainstem until the upper thoracic cord in a 55-year-old healthy woman following herpes zoster infection in the left C4-T3 dermatome. VZV-specific IgG in the cerebrospinal fluid (CSF) and AQP4-IgG positivity on enzyme-linked immunosorbent assay (ELISA) were undetectable. Thus, she was diagnosed with immune-competent VZV radiculomyelitis. Forty-two months later, she experienced a relapse, and AQP4-IgG positivity was detected on ELISA. A cell-based assay (CBA) showed AQP4-IgG positivity not only at the time of recurrence but also retrospectively at 1 month after the initial symptoms. We concluded that AQP4-IgG-positive neuromyelitis optica spectrum disorder (NMOSD) was concurrent with VZV myelitis. After the second attack, she was treated with azathioprine and has had no relapse since then.

**Conclusion:** We reported a case of VZV radiculomyelitis with confirmed concurrent AQP4-IgG positivity. NMOSD induced by herpes zoster has been recently identified, but distinguishing it from VZV myelitis can be difficult and whether these two diseases aggravate each other is unknown. Awareness of the potentially varied presentation of VZV myelitis can enable earlier recognition and proper treatment.

## Introduction

Neuromyelitis optica (NMO; also known as Devic's disease) is considered an idiopathic inflammatory syndrome associated with severe attacks of optic neuritis and longitudinally extensive transverse myelitis (LETM), previously thought to be without brain involvement. Further advances have since been made due to the discovery of specific NMO-immunoglobulin-G (IgG)/aquaporin-4 (AQP4) antibody, the clinical and neuroimaging spectrum of NMO was further broadened by the International Panel for NMO Diagnosis (2015) to NMO spectrum disorder (NMOSD), wherein cerebral, brain stem, diencephalic lesions were also recognized with MRI. Both CNS lesions, and the presence of NMO-IgG/AQP4 antibody distinguish NMOSD from multiple sclerosis (MS). Moreover, the loss of AQP4 immunoreactivity and astrocyte pathology in the brain and spinal cord further discriminate NMOSD from MS.

The etiology of NMOSD is not fully understood. Serum and cerebrospinal fluid (CSF) analyses in patients with NMOSD suggest that autoimmune or infectious disorders possibly trigger disease onset (1). Among neurological or systemic autoimmune diseases, Sjogren's syndrome, systemic lupus erythematosus, myasthenia gravis, and thyroid disease are frequently reported to be associated with NMOSD (2). Regarding infectious diseases, *Helicobacter pylori* and *Chlamydia pneumoniae* infections are reported as risk factors for AQP4-IgG-positive NMOSD (3). Some reports have suggested that the development of NMO or AQP4-IgG-positive NMOSD is related to cytomegalovirus, herpes simplex virus, varicella-zoster virus (VZV), mumps, Epstein-Barr virus, and dengue virus (1, 4–6). Several reports have mentioned the relationship between NMO and VZV, but none of them investigated AQP4-IgG (7). Conversely, there have been few case reports that clearly outline the association between AQP4-IgG-positive NMOSD and herpes zoster infection (1, 8–11).

VZV myelitis, a rare disease that mostly affects immunocompromised patients and is caused by direct invasion of VZV of the spinal cord and occasionally, the spinal roots, presenting as radiculitis. In some cases, VZV myelitis is also observed in immunocompetent patients and described as LETM. The clinical course and long-term prognosis of immunocompetent patients are distinct from those of immunocompromised ones, perhaps because of the unforeseen effects of the immune process (12).

Distinguishing VZV myelitis from AQP4-IgG-positive NMOSD preceded by herpes zoster is critical for appropriate treatment. Although some studies have reported AQP4-IgG-positive NMOSD induced by herpes zoster infection, there is limited evidence on the co-occurrence of AQP4-IgG-positive NMOSD and VZV myelitis. Here, we report a case in which VZV radiculomyelitis co-existed with AQP4-IgG-positive NMOSD and review the relevant literature.

### Case Description

**Figure 1:**
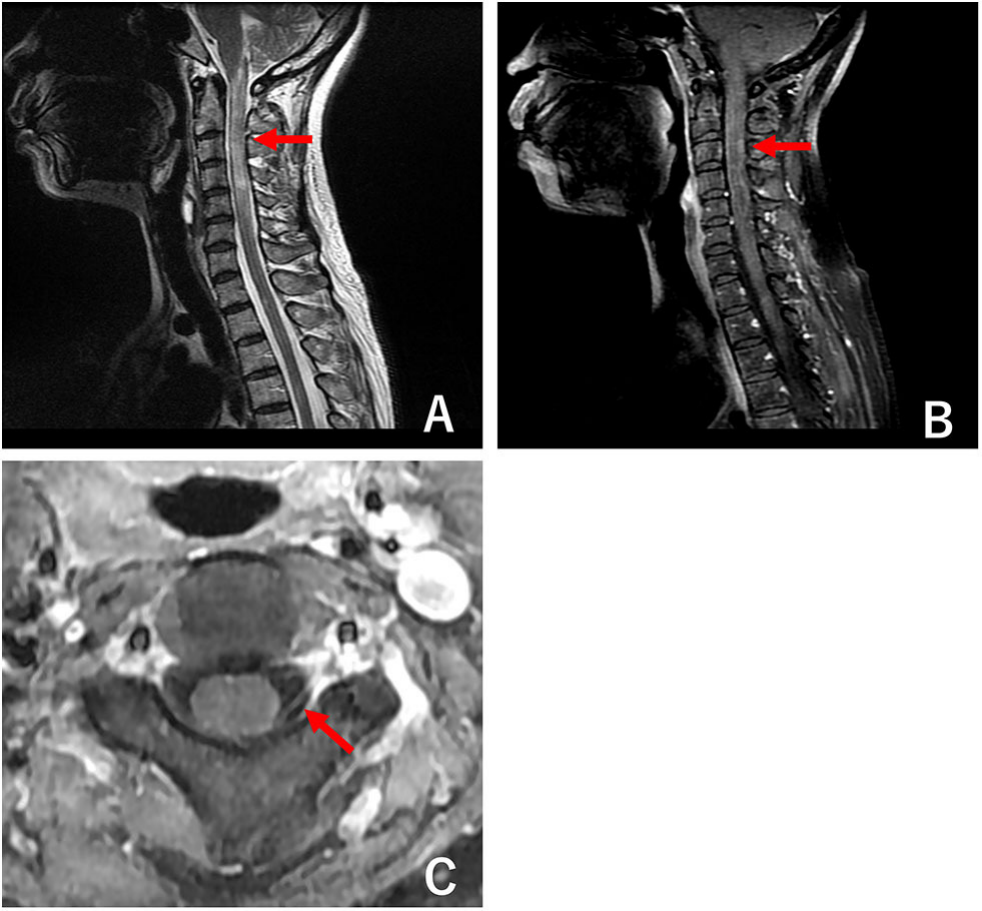
Magnetic resonance imaging (MRI) of cervical to thoracic spinal cord. **(A)** Sagittal T2 MRI showing longitudinally extensive transverse myelitis (arrow) from the lower part of the medulla oblongata to C5 with marked edema. **(B)** Gadolinium contrast MRI of cervical to thoracic spinal cord showing mild enhancement (arrow), which represents the active lesions of myelitis. **(C)** Axial gadolinium contrast MRI showing active radiculopathy of the left side (arrow).

A 55-year-old female complained of a pruritic skin lesion on the area involving the left C4-T3 dermatome. She visited a dermatologist who diagnosed dermatomal VZV infection and prescribed valaciclovir hydrochloride. Two weeks later, the skin lesion began to subside gradually, but she experienced bilateral numbness of the upper extremities, which progressively worsened, with development of dysesthesia of the right side of the face. A month after VZV infection, she was admitted to our hospital. Her medical history included gastroesophageal reflux disease and bilateral cataracts. Her physical examination revealed a minimal eschar near her left ear. Neurological examination showed dysesthesia of the right side of the face, neck, bilateral upper extremities, and T4-T10 levels. The examination of other cranial nerves was unremarkable. There was no note of muscle weakness or atrophy. Tendon reflexes were increased in all extremities with the Babinski reflex present bilaterally. Urine incontinence was also observed. Spinal magnetic resonance imaging (MRI) revealed LETM, extending from the lower part of the medulla oblongata to C5, with marked edema and moderate gadolinium enhancement (Figures 1A,B). Abnormal gadolinium enhancement of the left spinal posterior root was also observed (Figure 1C). Brain MRI showed no cranial lesions or optic neuritis. Antineutrophil cytoplasmic antibodies (ANCA, MPO, PR3) and antibodies against Sjögren syndrome-related antigens A and B were not detected. CSF analysis showed lymphocytic pleocytosis (50 cells/μl), increased total proteins (46.7 mg/dl), and negative oligoclonal bands. While VZV was undetectable on polymerase chain reaction (PCR), the VZV-specific antibody index was found to be increased (4.53; reference range: 0.7–1.5). Enzyme-linked immunosorbent assay (ELISA) of serum AQP4-IgG antibody yielded negative results. Therefore, she was diagnosed with VZV radiculomyelitis and treated with intravenous acyclovir 500 mg thrice daily for a week and methylprednisolone (IVMP) 1 g once daily for 3 days, followed by oral prednisolone (20 mg/day). The patient exhibited only a mild response to treatment.

**Figure 2:**
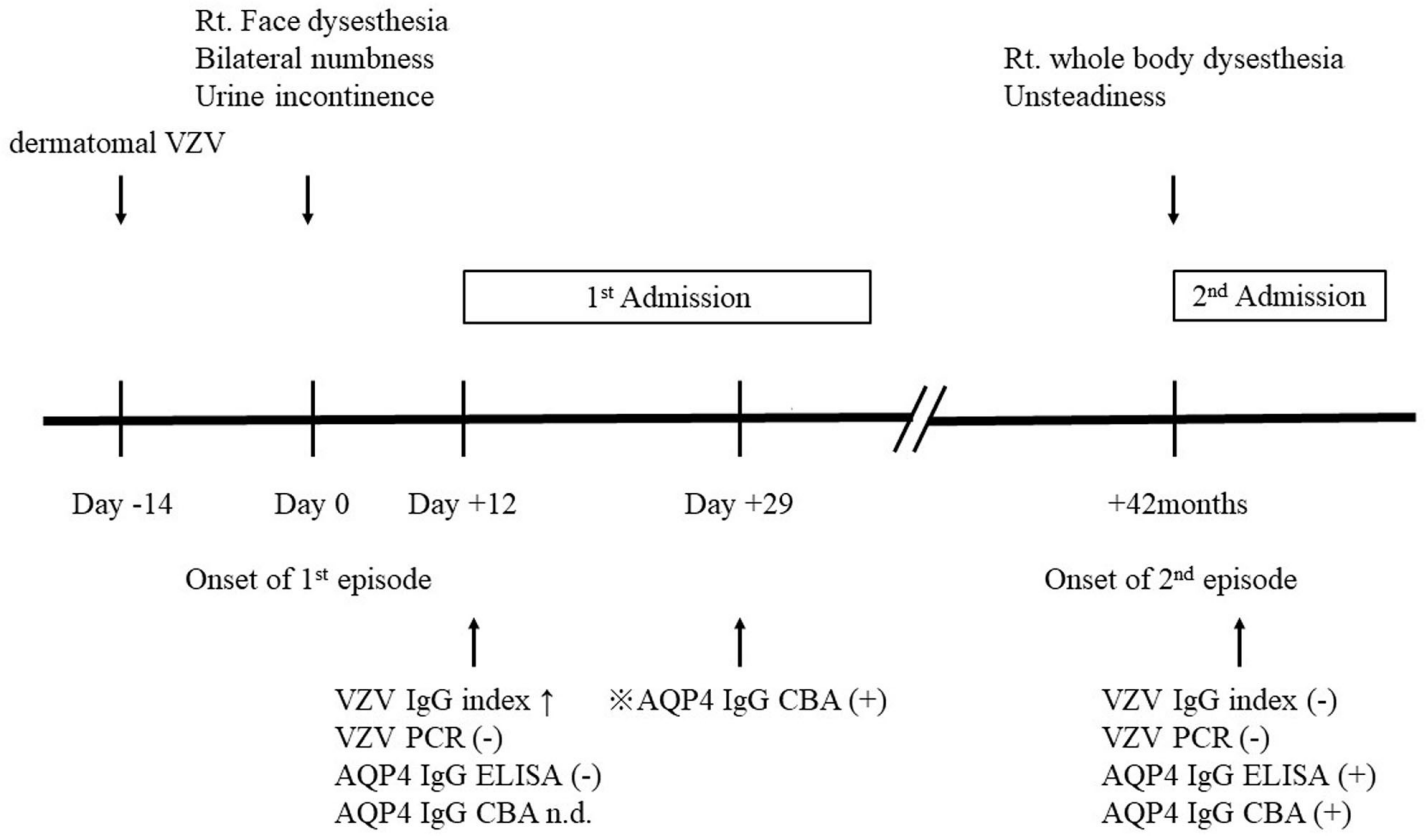
Timeline of patient's disease. Rt, right; VZV, varicella-zoster virus; n.d., not determined; AQP4-IgG, aquaporin-4 immunoglobulin-G; 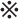 detected retrospectively.

The patient's second episode took place 42 months later. She experienced severe dysesthesia in her right hand, which gradually worsened after 2 days, and she started to experience right-sided dysesthesia for a second time. This was now accompanied by general unsteadiness. She had no skin lesions at this time. Upon consultation, ataxia with mild weakness in her right upper and lower extremities were noted. Spinal cord MRI showed a relapse of LETM in C3-T2, and CSF analysis revealed lymphocytic pleocytosis (30 cells/μl). VZV was undetected on PCR, and the VZV-specific antibody index was negative. However, the results of ELISA for serum AQP4-IgG were positive. We diagnosed her with AQP4-IgG-positive NMOSD and started her on IVMP 1 g once daily for 3 days, followed by oral prednisolone 40 mg/day, which was gradually tapered to 6 mg/day, and azathioprine 50 mg/day. These were continued and she experienced no relapse since. We theorized that she already had AQP4-IgG during the initial attack that went undetected. Thus, we tested the serum obtained during first admission using cell-based assay (CBA), and true enough, AQP4-IgG was detected (Figure 2). We concluded that VZV myelitis and AQP4-IgG-positive NMOSD co-existed in the patient during the initial events.

## Discussion

AQP4-IgG is a pathogenic marker of NMOSD. Asymptomatic AQP4-IgG seropositive status may exist for years before clinical NMOSD presentation (13); hence, AQP4-IgG-positive patients may need another “trigger” to develop the disease. Data show that approximately 15–35% of NMOSD patients had an infectious disease prior to NMOSD development. Several previous reports have shown a relation between NMO and VZV infection, including five AQP4-IgG-positive NMOSD cases preceded by herpes zoster infection (Table 1). Two of them showed VZV-positivity in the CSF during the onset of VZV myelitis, with AQP4-IgG being detected only during recurrence (9, 10). Two others showed VZV-negativity in the CSF and AQP4-IgG-positivity and they were diagnosed with AQP4-IgG-positive NMOSD preceded by herpes zoster (1, 7). Only one case of the five had both VZV positivity in the CSF and serum AQP4-IgG positivity similar to those seen in our case (8).

**Table 1:** Clinical features of para varicella zoster virus associated myelitis with aquaporin-4 immunoglobulin G positivity.

**References**	**Sex/age**		**MRI**	**CSF VZV studies**	**AQP4-IgG**	**Treatment**
Heerlein et al. (1)	F/63	1st	C7-T9 Gd pos. Rt. lateral ventricle Gd neg.	IgG index WNL PCR neg.	pos.	Acyclovir IVMP
Park et al. (9)	F/29	1st	Rt. lower midbrain Gd neg.	IgM inc. PCR neg.	n.d.	Ventilator support Plasma exchange
		2nd	C2-C7 Gd pos.	IgG index n.d. PCR n.d.	pos.	IVMP
Machado et al. (10)	F/77	1st	C2-T12 Gd neg.	IgG index n.d. PCR pos.	n.d.	Acyclovir Prednisolone
		2nd	Cerebellum Gd neg.	IgG index n.d. PCR neg.	pos.	Steroids Azathioprine
Mathew et al. (11)	F/48	1st	C2-C5 Gd n.d.	IgG index n.d. PCR neg.	pos.	Acyclovir IVMP
Suda et al. (8)	F/53	1st	T1-T7 Gd pos. Rt. lateral ventricle Gd neg.	IgG index inc.	pos.	Acyclovir IVMP
		2nd				
Present case	F/55	1st	Medullar oblongata-C5, Lt. C5, C6 nerve root, T2-T10 Gd pos.	IgG index inc. PCR neg.	pos.	Acyclovir IVMP Prednisolone
		2nd	C3-T2 Gd pos.	IgG index WNL PCR neg.	pos.	IVMP Prednisolone Azathioprine

*F, female; Rt, right; Lt, left; Bil, bilateral; neg., negative; pos., positive; inc., increase; WNL, within normal limits; Gd, gadolinium enhancement; VZV, varicella-zoster virus; n.d., not determined; IVMP, methylprednisolone pulse therapy; AQP4-IgG, aquaporin-4 immunoglobulin-G*.

In our patient, during the initial attack, LETM from the medulla to the cervical spine led to the suspicion of NMOSD. However, AQP4-IgG was undetectable on ELISA, and the VZV antibody index was found to be increased in the CSF. Moreover, MRI revealed radiculomyelitis corresponding to the contiguous zoster dermatomes. Consequently, we diagnosed the patient with VZV radiculomyelitis and did not further examine AQP4-IgG using the highly sensitive CBA, which requires a specialized laboratory set-up. However, during recurrence 42 months later, the patient was found to have seroconverted to AQP4-IgG positivity and was thus diagnosed with AQP4-IgG-positive NMOSD. Based on the positive result of the retrospective examination of AQP4-IgG by CBA during the initial attack, we concluded that NMOSD co-existed with VZV radiculomyelitis.

There is also growing evidence suggesting that the clinical course and long-term prognosis of VZV myelitis may be different in immunocompromised and immunocompetent patients. A study suggested that immunocompetent patients with VZV myelitis were mostly women, aged 18–47 years, with myelitis in the cervical or thoracic spinal cord and with benign disease courses. These characteristics were consistent with those of AQP4-IgG-positive NMOSD. In some cases, these immunocompetent patients did not show anti-VZV antibody and VZV DNA in the CSF, and data on AQP4-IgG were not acquired (14). We speculate that these immunocompetent VZV myelitis cases may be complicated by AQP4 IgG-positive NMOSD. In our case, we could not detect AQP4 IgG, possibly due to low sensitivity tests; this may hold true for CBA as well. Further studies are warranted to elucidate the exact role of concomitant elevation of AQP4-IgG in VZV myelitis, and its evolution to NMOSD (12, 15).

The pathophysiology of concurrence between VZV myelitis and AQP4-IgG is not known. Previously, NMO was attributed to VZV (7), and the triggering role of infectious events could be explained by molecular mimicry and bystander activation. Molecular mimicry is the activation of B-cells producing antibodies that recognize both the microbial and the self-AQP4 epitopes. Alternatively, bystander activation involves damage to the AQP4-rich tissue by microbes, leading to the activation of AQP4-specific T and B-cells. In our case, we hypothesize that AQP4-abundant spinal cord tissue damage was due to the direct invasion of VZV activated AQP4-IgG. Otherwise, other mechanisms, such as blood-brain barrier breakdown, which allows the autoantibody to cross the blood-brain barrier, may play a role in the production of AQP4-IgG.

Our case report has some limitations. Our case was diagnosed clinically and without histopathologic documentation of direct invasion of VZV in the spinal cord, which are mostly observed in immunocompromised patients with VZV myelitis (16). To prove the coexistence of VZV myelitis and AQP4-IgG-positive NMOSD, a comprehensive workup in immunocompetent patients with VZV myelitis is recommended. In future studies, we need to investigate the frequency of activated AQP4-IgG in patients with herpes zoster or VZV myelitis, in a large cohort. A mouse model with VZV infection or VZV myelitis may be used to check for the presence of AQP4-IgG and possible concurrence of NMOSD myelitis pathology.

In conclusion, while the diagnostic criteria of NMOSD requires exclusion of other diseases, we should take into account that concurrence of VZV radiculomyelitis with AQP4-IgG-positive NMOSD can occur and thereby alter the disease course. Further, we need to know the frequency of these coexistences. Therefore, when VZV myelitis is diagnosed, repeated examinations for AQP4-IgG are recommended.

## Ethics Statement

Written informed consent was obtained from the individual for the publication of any potentially identifiable images or data included in this article.

## Author Contributions

HE performed the data research and wrote the paper. HT and SN treated the patient. MK, AN, AF, and JF supported the clinical interpretation. HM and YS were critically involved in the theoretical discussion and composition of the manuscript. All authors read and approved the final version of the manuscript.

## Conflict of Interest

The authors declare that the research was conducted in the absence of any commercial or financial relationships that could be construed as a potential conflict of interest.
